# Absence of *in vivo* mutagenicity of multi-walled carbon nanotubes *in* single intratracheal instillation study using F344 *gpt* delta rats

**DOI:** 10.1186/s41021-016-0065-5

**Published:** 2017-01-06

**Authors:** Katsuyoshi Horibata, Akiko Ukai, Akio Ogata, Dai Nakae, Hiroshi Ando, Yoshikazu Kubo, Akemichi Nagasawa, Katsuhiro Yuzawa, Masamitsu Honma

**Affiliations:** 1 Division of Genetics and Mutagenesis, National Institute of Health Sciences, 1-18-1 Kamiyoga, Setagaya, Tokyo, 158-8501 Japan; 2 Department of Pharmaceutical and Environmental Sciences, Tokyo Metropolitan Institute of Public Health, 3-24-1 Hyakunin-cho, Shinjuku, Tokyo, 169-0073 Japan; 3 Present address: Department of Nutritional Science and Food Safety, Faculty of Applied Bioscience, Tokyo University of Agriculture, 1-1-1 Sakura-ga-Oka, Setagaya, Tokyo, 156-8502 Japan

**Keywords:** Carbon nanoparticle, in vivo genotoxicity, *Pig-a* gene mutation assay, Multi-walled carbon nanotubes

## Abstract

**Introduction:**

It is known that fibrous particles of micrometer length, such as carbon nanotubes, which have same dimensions as asbestos, are carcinogenic. Carcinogenicity of nanomaterials is strongly related to inflammatory reactions; however, the genotoxicity mechanism(s) is unclear. Indeed, inconsistent results on genotoxicity of multi-walled carbon nanotubes (MWCNTs) have been shown in several reports. Therefore, we analyzed the in vivo genotoxicity induced by an intratracheal instillation of straight MWCNTs in rats using a different test system—the *Pig-a* gene mutation assay—that can reflect the genotoxicity occurring in the bone marrow. Since lungs were directly exposed to MWCNTs upon intratracheal instillation, we also performed the *gpt* assay using the lungs.

**Findings:**

We detected no significant differences in *Pig-a* mutant frequencies (MFs) between the MWCNT-treated and control rats. Additionally, we detected no significant differences in *gpt* MFs in the lung between the MWCNT-treated and control rats.

**Conclusions:**

Our findings indicated that a single intratracheal instillation of MWCNTs was non-mutagenic to both the bone marrow and lung of rats.

## Introduction

Nanomaterials are important substances in nanotechnology, and potential risks to humans and the environment need to be investigated for risk assessment and management. There are several reports on the toxicities induced by carbon nanoparticles, such as fullerene (C_60_), single-walled carbon nanotubes, and multi-walled carbon nanotubes (MWCNTs). It is known that fibrous or rod-shaped particles of micrometer length, which share the dimensions with asbestos, are carcinogenic to humans and experimental animals [1–5]. The carcinogenicity and toxicity induced by exposure to nanomaterials are strongly related to inflammatory reactions and reactive oxygen species [6–10]. In particular, mesothelioma was induced by intraperitoneal application of MWCNTs in p53^+/−^ mice [4] and by intrascrotal administration of MWCNTs to wild-type rats [5]. Additionally, malignant mesothelioma and lung tumors were induced by administration of MWCNT to the lung via the trans-tracheal intrapulmonary spraying method [11]. However, the genotoxicity mechanism(s) related to carcinogenesis induced by MWCNT treatment is unclear.

There are several reports on the in vivo genotoxicity of MWCNTs, but they provide conflicting data [12–15]. In vivo comet assay revealed that a single intratracheal or pharyngeal instillation of MWCNTs to mice both induced DNA damage in the lungs in a dose-dependent manner [13, 15]. However, another group reported that MWCNTs administered by gavage showed no genotoxic effect in an in vivo micronucleus test [12]. Additionally, an intratracheal instillation of MWCNTs to rats was also non-genotoxic, as demonstrated by the comet assay of the lung [14]. Using a different endpoint, it was reported that an intratracheal instillation of MWCNTs in mice increased the mutation frequency detected by the *gpt* assay in the lung [13].

These discrepancies prompted us to examine in vivo mutagenicity in the lung, induced by an intratracheal instillation of MWCNTs, using *gpt*-delta transgenic rats because the lung was directly exposed to MWCNTs under our experimental conditions. The *gpt* assay is known as one of the gene mutation assays using a transgenic rodent. Additionally, we employed a different test system, the recently established *Pig-a* gene mutation assay [16–19]. This is a powerful tool for the evaluation of in vivo genotoxicity and is based on flow cytometric enumeration of glycosylphosphatidylinositol (GPI) anchor-deficient erythrocytes [16, 17]. It is applicable across species, from rodents to humans [20–24]. For this method, no transgenic animals are needed to test in vivo genotoxicity; all that is needed is a small volume of peripheral blood [16, 17]. Additionally, long-term, accumulated in vivo genotoxic effects could be evaluated by the *Pig-a* assay [25].

## Materials and methods

### Animals

F344/NSlc-Tg (*gpt*-delta) male rats were obtained from Japan SLC (Shizuoka, Japan). The animals were housed individually under specific pathogen-free conditions with a 12-h light–dark cycle. Food (CRF-1 pellet feed, Oriental Yeast Co., Ltd., Tokyo, Japan) and water were available *ad libitum*. Animal experiments were conducted in accordance with the regulations of the Animal Care and Use Committees of the National Institute of Health Sciences and Tokyo Metropolitan Institute of Public Health.

### Test chemicals

MWCNTs (Lot No. 060125-01 k, Mitsui & Co., Ltd., Ibaraki, Japan) were prepared as described previously [4, 5, 26], with some modifications. According to the reports, these straight MWCNT fibers are approximately 100 nm in diameter, and 27.5% of the MWCNTs are ≥ 5 μm in length. The MWCNTs were suspended in a 0.2% (w/v) carboxymethyl cellulose (CMC) solution (Kanto Chemical Co., Ltd., Tokyo, Japan). The suspensions and the vehicle (0.2% CMC solution) were sterilized in an autoclave at 120 °C for 20 min and vigorously mixed by hand shaking immediately prior to the administration [5]. *N*-nitroso-*N*-ethylurea (ENU, Sigma) was dissolved in phosphate-buffered saline (pH 6.0) at 10 mg/mL as described previously [18].

### Antibodies

We obtained anti-rat CD59 [clone TH9, fluorescein isothiocyanate (FITC)-conjugated] and anti-rat erythroid marker (clone HIS49, allophycocyanin-conjugated) antibodies from BD Biosciences (Tokyo, Japan).

### Dose levels and treatments

At eight weeks of age, six male rats per group received a single intratracheal treatment of MWCNTs (0.25, 0.5, or 1 mg/kg) or the vehicle (negative control). The intratracheal spraying injection was performed using a microsprayer (series IA-1B intratracheal aerosolizer; Penn-Century, Philadelphia, PA, USA) as described previously [27]. ENU (35.6 mg/kg) was administered intraperitoneally to six male rats for the positive control. Additionally, six male rats were treated with MWCNTs (intratracheally, 1 mg/kg) plus ENU (intraperitoneally, 35.6 mg/kg). At four weeks after the administration, all rats were sacrificed, and blood and lung samples were collected for the *Pig-a* and *gpt* assays, respectively. Because of the high cost of the *gpt* assay, the lung samples of both the control groups and highest dose group were analyzed prior to the other groups. In the case where a negative genotoxicity was detected in the highest dose group, analyses of the other groups were discontinued.

### *Pig-a* mutation assay

The *Pig-a* assay was performed as described previously [28, 29]. Blood (3 μL) was labeled with anti-rat CD59 (1 μg) and anti-rat erythroid marker (0.133 μg) antibodies. Approximately 1 × 10^6^ erythroid marker-positive cells were analyzed using a FACSCanto II flow cytometer (BD Biosciences) for the presence of surface CD59, and *Pig-a* mutant frequencies (MFs) were expressed as the number of CD59-negative cells per one million of HIS49-positive red blood cells (RBCs). To avoid artifactually inflating *Pig-a* MFs, we refined the gate for *Pig-a* mutant RBCs as the area encompassing a maximum of 99.0% of the lower RBC FITC staining intensities only, as previously described [28–31].

### *gpt* mutation assay

High-molecular-weight genomic DNA was extracted from the lung samples of the rats treated with MWCNTs (1 mg/kg), the vehicle, and ENU using a Recover Ease DNA isolation kit (Agilent Technologies, Santa Clara, CA, USA). Lambda EG10 phages were rescued using the Transpack packaging extract (Agilent Technologies), and the *gpt* mutation assay was conducted as described previously [28, 32]. *gpt* MFs were calculated by dividing the number of confirmed 6-thioguanine-resistant colonies by the number of colonies with rescued plasmids [32].

### Calculations and statistical analyses

Statistical analyses were performed using Excel Statistics 2012 (Social Survey Research Information, Tokyo, Japan) as follows. Distributions were tested by the Bartlett’s test. If the distributions were normal, one-way analysis of variance was applied, followed by the Dunnett’s post-hoc test (pairwise comparisons of the frequencies in the treated groups to that in the vehicle control group, one-sided). Otherwise, the Kruskal–Wallis test was applied for analysis, followed by the Steel’s post-hoc test (pairwise comparisons of the frequencies in the treated groups to that in the vehicle control group, one-sided).

## Results

### *Pig-a* assay

**Fig. 1:**
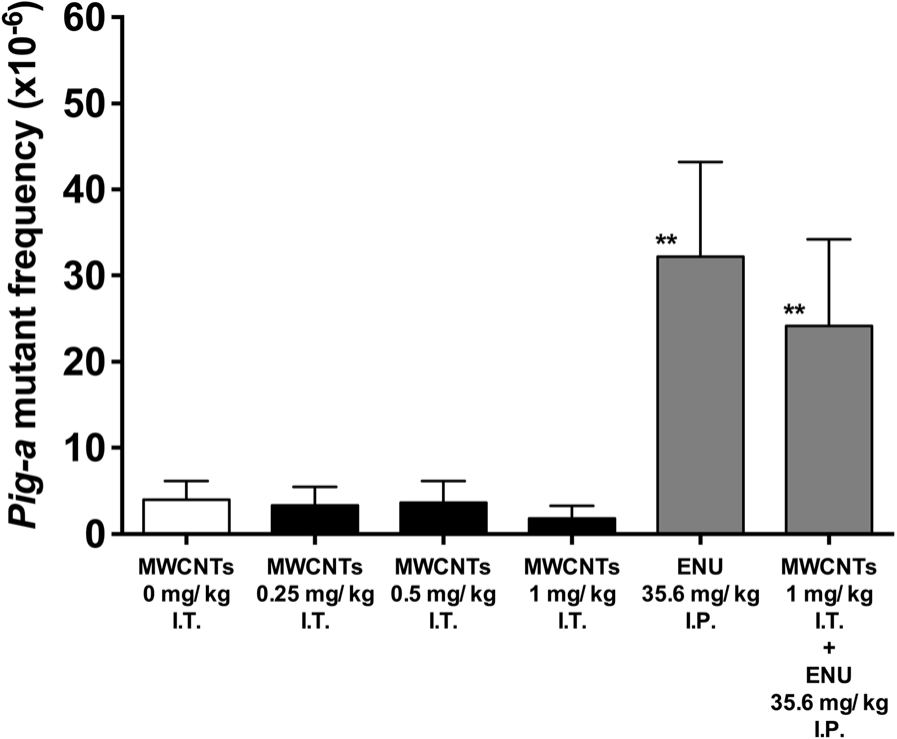
*Pig-a* mutant frequencies. Four weeks after the treatments, blood was withdrawn and analyzed by flow cytometry for the presence of surface CD59 on RBCs. Each treated group is shown under the bars. The data are the mean ± standard deviation (SD). **p < 0.01 compared to the control

Compared with the control, the *Pig-a* MFs clearly increased in the ENU- and ENU plus MWCNT-treated rats (Fig. 1). In contrast, we detected no significant differences in the *Pig-a* MFs among the MWCNT-only-treated groups (Fig. 1). Additionally, we detected no significant differences in the *Pig-a* MFs between the ENU- and ENU plus MWCNT-treated rats (Fig. 1).

### *gpt* assay

**Fig. 2:**
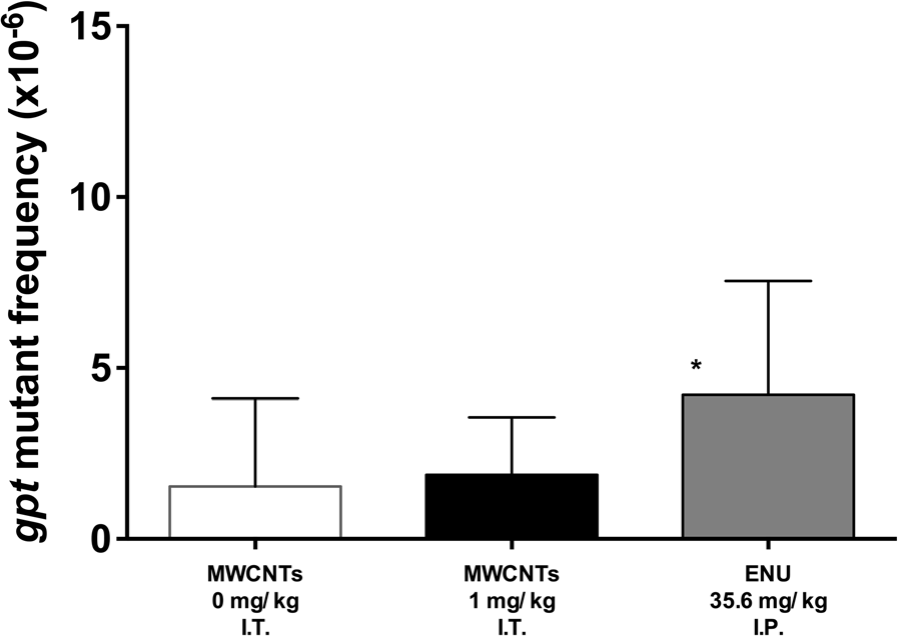
*gpt* mutant frequencies in the lung. Four weeks after the treatments, the rats were sacrificed, and their lung samples were collected and analyzed by the *gpt* assay. Each treated group is shown under the bars. The data are the mean ± SD. *p < 0.05 compared to the control

Compared with the control, the *gpt* MFs significantly increased in the ENU-treated rats (Fig. 2 and Table 1). In contrast, we detected no significant differences in the *gpt* MFs in the lungs among the MWCNT-treated groups (Fig. 2 and Table 1).

**Table 1:** Induction of mutations (*gpt* assay) in the lungs of transgenic rats treated with MWCNTs

Compound	Dose (mg/kg)	Animal No.	Colony-forming units	Mutants	Mutant frequency (×10^−6^)
0.2% CMC^*^	0	1	756,000	5	6.61
		2	1,284,000	2	1.56
		3	924,000	0	0.00
		4	720,000	0	0.00
		5	939,000	1	1.06
		6	621,000	0	0.00
		Ave.			1.54
		S.D.			2.57
MWCNT	1	1	1,035,000	2	1.93
		2	810,000	4	4.94
		3	930,000	0	0.00
		4	909,000	2	2.20
		5	951,000	1	1.05
		6	870,000	1	1.15
		Ave.			1.88
		S.D.			1.69
ENU	35.6	1	777,000	1	1.29
		2	894,000	4	4.47
		3	744,000	3	4.03
		4	510,000	1	1.96
		5	567,000	6	10.58
		6	678,000	2	2.95
		Ave.			4.21**
		S.D.			3.34

*CMC, a carboxymethyl cellulose solution (negative control)

**p < 0.05 compared to the control

## Discussion

It has been reported that aspiration exposure of mice to MWCNTs induced strong inflammatory reactions [33], and an intratracheal instillation of MWCNTs to rats resulted in strong pulmonary toxicity effects, including focal peribronchiolar lymphoid aggregates, foamy alveolar macrophage accumulation, lymphoplasmocytic infiltration, fibrosis, and diffuse alveolar damage [34]. Inflammatory reactions induced by MWCNTs treatment seem to be the cause of genotoxicity [13].

An in vivo comet assay revealed that a single intratracheal instillation of MWCNTs to mice induced DNA damage in the lungs in a dose-dependent manner. Oxidative DNA damage related to inflammation was also detected in mouse lungs [13]. Additionally, the in vivo comet assay using mouse lungs revealed that single pharyngeal aspiration of straight MWCNTs, but not tangled MWCNTs, induced DNA damage in a dose-dependent manner [15]. However, oral administration of MWCNTs to mice showed no genotoxicity in the erythrocyte micronucleus test [12]. Both single and repeated doses of intratracheally instilled MWCNTs were found by comet assays to be non-genotoxic to rat lung cells, although inflammatory changes, including infiltration of macrophages and neutrophils, were detected in the rats [14]. In the above reports, in vivo genotoxicity of MWCNTs was evaluated by direct detection of DNA damage, e.g., using the comet assay and micronucleus test. However, the results were inconsistent. To elucidate the in vivo genotoxicity potential of MWCNTs, we employed different endpoints and evaluated in vivo genotoxicity of MWCNTs using two gene mutation assays, i.e., the *Pig-a* and *gpt* assays.

We demonstrated that straight MWCNTs administered intratracheally to male F344/NSlc-Tg (*gpt*-delta) rats were negative for genotoxicity in the *Pig-a* assay using blood (Fig. 1). The *Pig-a* assay is based on the detection of a GPI-anchored protein on the cell surface of RBCs. It is considered that the absence of the GPI-anchored protein in RBCs is caused by mutations in the *Pig-a* gene, occurring in nucleated erythroid precursors and/or in hematopoietic stem cells (HSCs) [16, 19]. This suggests that the expression of GPI-anchored CD59 in RBCs depends on the *Pig-a* mutations that occurred in erythroid precursors and/or HSCs in the bone marrow. According to this, we considered that our results obtained by the *Pig-a* assay reflected the genotoxicity of MWCNTs to the bone marrow. We also detected no significant difference in the *Pig-a* MFs between the groups treated with ENU alone or with ENU plus MWCNTs. This result indicated that the treatment with MWCNTs did not increase the ENU genotoxicity to the bone marrow. Because the target organ was the lung in our experiments, the negative results of the *Pig-a* assay were not unexpected. On the other hand, MWCNTs were non-genotoxic to the lungs as shown by the *gpt* assay, although the lungs were directly exposed to MWCNTs (Fig. 2). Based on these findings, we concluded that no mutagenicity was induced by a single intratracheal treatment with straight MWCNTs in both the lung and bone marrow. Interestingly, similar results were shown by Kato et al. [13]. In their report, the *gpt* assay revealed that no genotoxicity was induced by a single- and two-times repeated intratracheal instillation; however, a four-times repeated treatment resulted in genotoxicity.

All these studies indicate that the nature of MWCNTs, exposure conditions, method, route, and endpoints are important when evaluating the genotoxicity of MWCNTs. At this time, we cannot explain the mechanism(s) of MWCNT genotoxicity in detail, but we suspect that the mechanism(s) is complex and depends on the nature of MWCNTs, oxidative DNA damages, inflammation, and other biological factors, as previously discussed for another nanomaterial, C_60_ [18].

## Abbreviations

C_60_: Fullerene
CMC: Carboxymethyl cellulose
ENU: *N*-nitroso-*N*-ethylurea
FITC: Fluorescein isothiocyanate
GPI: Glycosylphosphatidylinositol
HSC: Hematopoietic stem cell
MF: Mutant frequency
MWCNTs: Multi-walled carbon nanotubes
RBC: Red blood cell
